# In vitro performance of an autocured universal adhesive system in bonding to dentin

**DOI:** 10.1186/s12903-023-03645-0

**Published:** 2023-11-27

**Authors:** Kota Kibe, Takashi Hatayama, Yasushi Shimada

**Affiliations:** Division of Oral Health Sciences, Department of Cariology and Operative Dentistry, Graduate School of Medical and Dental Sciences, Tokyo Medical and Dental University, 1-5-45, Yushima, Bunkyo-Ku, Tokyo, 113-8510 Japan

**Keywords:** Universal adhesive, Autocured bonding, Optical coherence tomography, Confocal laser scanning microscopy, Marginal adaptation, Bond strength

## Abstract

**Background:**

The successful integration of resin-based dental adhesives significantly impacts restorative dentistry, providing efficient and aesthetically pleasing caries treatments. Among various adhesives, one-step self-etching adhesives (1-SEAs) have gained popularity due to their simplicity and short application time. However, concerns have been raised regarding their bonding performance and marginal adaptation characteristics, which differ from two-step self-etching adhesives (2-SEAs) and three-step etch-and-rinse adhesives. Additionally, light-cured bonding materials may encounter challenges in deep cavities and inaccessible areas, necessitating extended light irradiation time. Autocured bonding materials are a potential solution, but limited comparative studies have been conducted on their performance.

**Methods:**

In this in vitro study, we evaluated a new autocured universal bonding material (Bondmer Lightless 2) and compared the results with recent light-cured bonding materials. Microshear bond strength (μSBS) tests were performed on 25 human molars using five different combinations of adhesives and composite resins: Bondmer Lightless 2 with Estelite Bulk Fill Flow (BE group), Bondmer Lightless 2 with a prototype composite resin (BO group), Prime&Bond Universal with SDR flow + (PS group), Scotchbond Universal with Filtek Bulk Fill (SF group), and G-Premio Bond with Gracefil BulkFlo (GG group). The bond strengths and failure modes were assessed using a universal testing machine and scanning electron microscope (SEM), respectively. Marginal adaptation was evaluated using swept-source optical coherence tomography (SS-OCT) and confocal laser scanning microscopy (CLSM) on 40 sound bovine maxillary incisors.

**Results:**

The μSBS test showed no significant differences in bond strength among the tested groups. Most failure modes were observed at the bond interface between the adhesive and the dentin. The autocured bonding material demonstrated significantly higher marginal adaptation (SI%) than PS, SF, and GG. The CLSM images corresponded with gaps observed in the SS-OCT images, indicating improved marginal sealing in the autocured group.

**Conclusions:**

The new autocured universal bonding material exhibited comparable bond strength to a conventional light-cured material while demonstrating a superior marginal adaptation level. This finding suggested that the autocured material could be a valuable alternative, especially when extended light irradiation would pose a challenge. Further clinical studies would be warranted to evaluate the performance of the autocured bonding material in actual restorative dental practice.

## Introduction

The advent of resin-based dental adhesives has minimized the intervention of caries treatment, revolutionizing restorative dentistry. Various adhesives have evolved over the past several decades into complex formulations and simplified clinical procedures. Due to the simplicity and short application time of the technique, one-step self-etching adhesives (1-SEAs) have become more popular than two-step self-etching adhesives (2-SEAs) and three-step adhesives in recent years. When 1-SEA is applied, smear plugs remain, and postoperative sensitivity issues are significantly reduced. However, in vitro, certain 1-SEAs have lower bond strengths and higher spontaneous fracture rates than specific 2-SEAs. Clinical reports have shown that 3-step etch-and-rinse adhesives and 2-SEAs perform in good, reliable and predictable manners, while 1-SEA performs inefficiently, indicating that the adhesive performance of 1-SEA is controversial [1–3].

The marginal adaptation of a direct composite resin restoration is a key factor for achieving good clinical results. Composite resin restorations suffer from polymerization shrinkage [4, 5]. The shrinkage stress at the tooth–restoration interface can weaken the integrity of the restoration and form gaps. In addition, the poor adhesive performance of the bonding agent and the insufficient copolymerization of the adhesives [6, 7] may degrade marginal adaptation. Gaps and lack of continuity at the tooth–adhesive interface caused by these factors can affect the success of bonded restorations. Gaps at the interface can lead to bacterial microleakage [8], secondary caries, restored tooth sensitivity [9], debonding [10], and ultimately treatment failure [11, 12]. Therefore, marginal adaptation, which correlates with bond strength [13], and adequate polymerization of the bonding agent are important factors in direct composite resin restorations. Optical coherence tomography (OCT) has been proposed as a new nondestructive technique for producing micron-scale, high-resolution and cross-sectional images of internal biological structures [14]. Recently, this technique has been applied in dentistry for the characterization of caries [15], the evaluation of gaps at the composite–tooth interface in two-dimensional (2D) and three-dimensional (3D) images [16, 17] and the assessment of voids and internal defects in restorations [18]. Several scholars have used OCT to evaluate the marginal adaptation levels of restorations [16, 19–21]. Additionally, scholars have assessed the cavity fit of restorations using OCT for correlation using confocal laser scanning microscopy (CLSM) [22] and scanning electron microscopy (SEM) [23].

In recent years, most one-step bonding materials have been light-cured; chemical-cured (autocured) bonding materials are not common. Light-cured bonding materials are widely used due to the following advantages. Polymerization does not start until light irradiation; thus, the operation time can be freely adjusted. Furthermore, light is irradiated from the surface, resulting in high polymerization and hardness at the subsurface. Finally, the level of temperature dependence is low; therefore, the polymerization rate is stable under the specified environmental temperature conditions.

However, light-cured bonding materials can reportedly encounter polymerization failures in deep cavities that are inaccessible to light or in molars where the use of irradiators is challenging [24]. To prevent these failures, increasing the light irradiation time is often necessary. However, in clinical situations, it is often difficult to submit patients, particularly children or those with temporomandibular joint disorders with limited mouth opening capabilities, to prolonged light irradiation. These limitations highlight the problems associated with light-cured bonding materials, including a decreased rate of polymerization in cavities and increased operation time required to compensate for these issues. In contrast, autocured bonding materials are a promising solution for addressing these problems, as the bonding materials can automatically polymerize regardless of the cavity condition. Recently, a new autocured universal adhesive system was developed for multipurpose usage by Tokuyama Dental (Tokyo, Japan). However, few researchers have compared the bond strength and marginal adaptation characteristics of autocured bonding materials with those of light-cured bonding materials. The aims of this study are to evaluate the bonding performance and marginal adaptation attributes of a new autocured universal bonding material (Bondmer Lightless 2, Tokuyama Dental) to dentin and compare them with those of recent light-cured materials.

## Materials and methods

### Microshear bond strength (μSBS) test

A total of 25 caries-free human third molars were collected from Tokyo Medical and Dental University hospital according to the protocol approved by the Institutional Review Board of Tokyo Medical and Dental University, Tokyo, Japan (D2013-022–02). All included teeth were extracted for reasons unrelated to the study and are so-called excess material. Inclusion criteria for teeth selection were the absence of caries and complete coronal and root development and the absence of root fracture and resorption, endodontic treatment. The teeth were stored at 4 °C in a0.1% thymol solution.

**Fig. 1:**
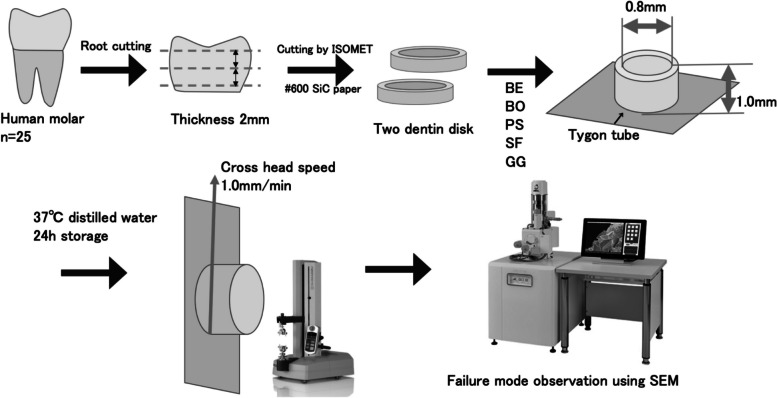
Sample preparation steps for the μSBS test

Sample preparation was performed as shown in Fig. 1. The roots were cut at the cementoenamel junction, and two dentin disks that were 2-mm-thick were cut for each tooth using a diamond blade (IsoMet, Buehler, Lake Bluff, IL, USA). The dentin disks were polished with water-resistant abrasive paper with grit levels reaching #600 under running water to flatten the surface. Fifty dentin disks were randomly assigned to one of the following 5 groups of 10 dentin disks each. All the materials were used in this study according to the manufacturers’ instruction. The materials used in this study are listed in Table 1 and below.

- BE group: Bondmer Lightless 2 was applied and air dried. Estelite Bulk Fill Flow was placed and photocured for 20 s. (Tokuyama Dental)
- BO group: Bondmer Lightless 2 was applied and air dried. a prototype composite resin developed for bulk filling, OCFB-001 was placed and photocured for 20 s. (Tokuyama Dental)
- PS group: Prim&Bond universal was applied for 20 s and photocured after air blowing. SDR flow + was applied and photocured for 20 s. (Dentsply, Konstanz, Germany)
- SF group: Scotchbond Universal was applied for 20 s and photocured after air blowing. Filtek Bulk Fill was applied and photocured for 20 s. (3 M ESPE, St. Paul, MN, USA)
- GG group: G-Premio Bond was applied for 10 s and photocured after air blowing. Gracefil BulkFlo was applied and photocured for 20 s. (GC, Tokyo, Japan)

**Table 1:** The materials used in this study

Code		Material	Compositions		Filler content (wt%)	Application protocol	Manufacturer
BE	Bond	Bondmer Lightless II	Bottle A	acetone, phosphate monomer, Bis-GMA, TEGDMA, HEMA, MTU-6, etc		rubbing for 1 ~ 10 s & air blow	Tokuyama Dental, Tokyo, Japan
			Bottle B	acetone, ethanol, water, borate catalyst, peroxide, silane coupling material, etc			
	Resin	Estelite Bulk Fill Flow (U)	silica-zirconia filler, composite filler, Bis-GMA, TEGDMA, Bis-MPEPP, mequinol, dibutyl hydroxyl toluene, UV absorber		70		
BO	Bond	Bondmer Lightless II	same			same	Tokuyama Dental, Tokyo, Japan
	Resin	OCFB-001	silica-zirconia filler, composite filler, UDMA, TEGDMA, camphorquinone, UV absorber		69		
PS	Bond	Prime & Bond universal	phosphoric acid modified acrylate resin, multifunctional acrylate, bifunctional acrylate, acidic acrylate, isopropanol, etc			rubbing for 20 s & light curing for 10 s	Dentsply, Konstanz, Germany
	Resin	SDR flow + Bulk Fill Flowable (U)	modified UDMA, TEGDMA, camphorquinone, ethyl-4(dimethylamino)benzoate photoaccelerator, etc		70.5		
SF	Bond	Scotchbond Universal Plus	brominated dimethacrylate, HEMA, phosphor-ylated methacrylate, ethyl alcohol, silane treated silica, etc			rubbing for 20 s & light curing for 10 s	3 M ESPE, St.Paul, MN, USA
	Resin	Filtek Bulk Fill Flowable Restorative (U)	Bis-GMA, TEGDMA, BisEMA, procrylat resins, ytterbium trifluoride filler, zirconia-silica filler, etc		64.5		
GG	Bond	G-Premio Bond	water, 4-methacryloxyethyl trimellitic acid, phosphate monomer, thiophosphate monomer, methacrylic ester, acetone			rubbing for 1 ~ 10 s & light curing for 10 s	GC, Tokyo, Japan
	Resin	Gracefil BulkFlo (U)	barium glass filler, Bis-MEPP		70		

*Bis-GMA* Bisphenol A-glycidyl methacrylate, *TEGDMA* Triethylene glycol dimethacrylate, *HEMA* 2-Hyroxyethylmethacrylate, *MTU-6* 6-Methacryloyloxyhexyl 2-thiouracil-5-carboxylate, *Bis-MPEPP* 2,2-Bis[(4-methacryloxy polyethoxy) phenyl] propane, *UDMA* Urethane dimethacrylate, *BisEMA* Ethoxylated bisphenol-A dimethacrylate, and *Bis-MEPP* Bismethacrylic acid isopropylidenebis (p-phenyleneoxyethylene) ester

We used a combination of a bonding system and a composite resin from the same manufacturer. For autocured bonding materials, BE and BO group were created. For light-cured bonding materials, PS, SF and GG group were created.

For sample preparation, Tygon tubes (R-3603, Norton Performance Plastics) of 0.8 mm in diameter and 1.0 mm in height were cut, two per disk were placed on the dentin disks with adhesives to fill the corresponding composite resin inside the tube space. As a result, two small resin cylinders of 0.8 mm in diameter and 1.0 mm in height for μSBS test were bonded to the dentin disks. The composite resin was light cured for 20 s (Valo LED Curing Light, high-power mode at 1,400 mW/cm^2^, Ultradent, South Jordan, UT, USA), and the tube was carefully removed from the resin composite cylinders. The specimens were stored in distilled water at 37 °C for 24 h before testing. The μSBS test was measured using a universal testing machine (EZ-Test-500N, Shimadzu, Kyoto, Japan) at a crosshead speed of 1.0 mm/min. The data obtained were statistically processed using one-way analysis of variance (ANOVA) at a significance level of 5%. After the μSBS test, the fracture surface was observed using SEM (JSM-5310LV, JEOL, Tokyo, Japan) to examine the failure mode. The area of each failure type (%) was calculated, and 70% of the surface was used as the threshold for classification. Failure modes were categorized into one of the following three types: cohesive failure of dentin, adhesive failure at the interface between adhesive and dentin, and mixed failure (combination of adhesive and cohesive failure). The results were statistically processed using the chi-square test. All analyses were performed using the Statistical Package for Social Science (SPSS; Windows, Version 23.0, IBM, Armonk, NY, USA).

### Swept-source optical coherence tomography (SS-OCT)

Cavity adaptation of each adhesive system was evaluated nondestructively using SS-OCT. The SS-OCT system (IVS-2000, Santec, Komaki, Japan) used in this study was a swept-source OCT. The system was incorporated with a low-coherence near-infrared light source and had the configuration of a Mach–Zehnder-type interferometer. The near-infrared light was produced by a fast sweeping laser that repetitively swept the wavelength from 1260 to 1360 nm (centred at 1310 nm) at a rate of 20 kHz. The axial resolution of this SS-OCT system in air was 11 μm, corresponding to 7 μm in dental tissue with a refractive index of approximately 1.5 [25]. The lateral resolution of the system was ∼17 μm, which was determined by the objective lens at the probe. The probe connected to the interferometer had a power of 5 mW, which was within the safety limit of the American Standard Institute. The laser source emitted from the probe was directed onto the sample at the desired location in the X and Z dimensions. The backscattered light carrying information from each single scan point of the sample was returned to the system, digitized on a time scale, and analysed in the Fourier domain to disclose the depth information (A-scan) of the sample. By combining a series of A-scans in a linear fashion across the sample, a cross-section (B-scan) was obtained. Finally, cross-sectional images could be created by converting the B-scan raw data into a greyscale image with 2001 × 1019 pixels.

### Specimen preparation

**Fig. 2:**
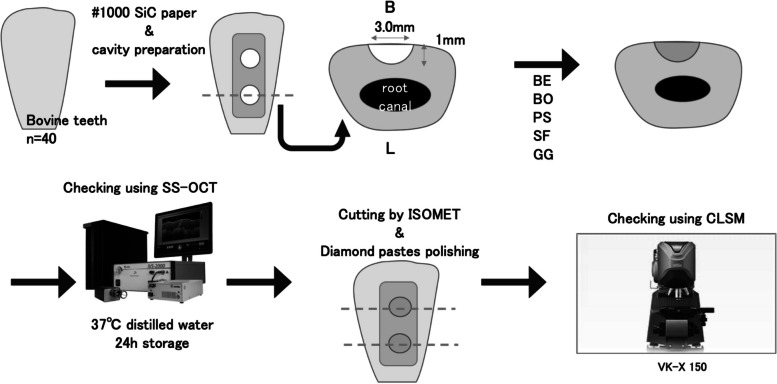
Sample preparation steps for analysis of the SS-OCT data

Sample preparation was performed, as shown in Fig. 2. A total of 40 sound bovine maxillary incisors were extracted for this study. The root was cut at the cementoenamel junction, and the labial enamel was polished with #1000-grit silicon carbide paper to remove the superficial layer and expose the dentin to form a flat surface. Class I dish-shaped cavities (1 mm in diameter × 1 mm in depth) were prepared using a diamond bar (#144 Diamond Point FG, Shofu, Kyoto, Japan) attached to a high-speed turbine handpiece under water cooling; thus, the margin was on the labial flat surface and the dentin cavity bottom. Two cavities per tooth were formed. For bonding operations, an adhesive system and composite of the aforementioned five groups were applied to the prepared cavities and cured according to the manufacturer's instructions. Then, each composite resin was filled and light-irradiated for 20 s (Valo LED Curing Light, high-power mode at 1,400 mW/cm^2^, Ultradent, South Jordan, UT, USA). The samples were stored in distilled water at 37 °C for 24 h.

### Analysis of SS-OCT data

**Fig. 3:**
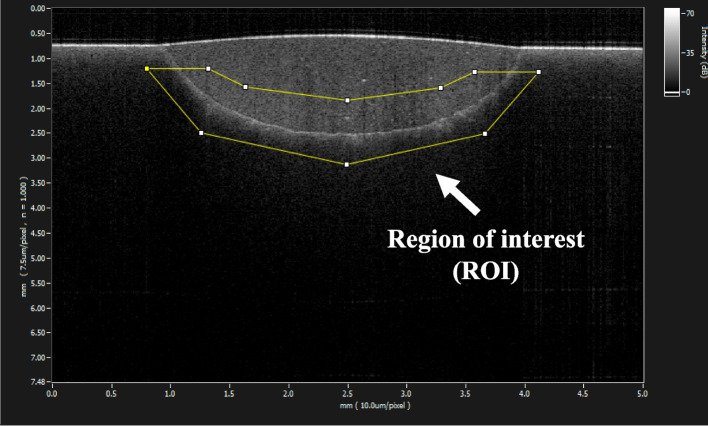
Location of the region of interest in the SS-OCT image. The ROI was selected and set near the entire length of the adhesive interface, excluding the sample surface

The samples were used for SS-OCT observation to capture the 2D images. Each sample was fixed to the microscale head stage of the SS-OCT, and the scanning laser beam was directed perpendicular to the repair surface. The sample was moved in a mid-range direction across the laser beam, and a cross-sectional B-scan image was captured at the maximum depth. When air was in a defect at the restoration–tooth interface, as the light passed between these two mediums with different refractive indices, the light partially reflected from the interface, which appeared as bright areas on the OCT image [13]. In this study, to analyse images, we adopted sealed interface percentage (SI%) parameters to calculate and evaluate the bonding performance levels of the adhesives. 2D SS-OCT raw tomograms were imported to image analysis software (ImageJ version 1.53t), and a median filter was applied to decrease background noise [26]. An experimental threshold determination algorithm developed as a plugin for ImageJ under JAVA was used for image analysis [22]. As shown in Fig. 3, the region of interest (ROI) was selected as a polygon around the whole length of the restoration interface, excluding the specimen surface. The width of the ROI was approximately 80 pixels, and the adhesive interface was placed in the centre of the ROI. The pixel values on each vertical line (A-scan corresponding to 2 pixels in width) in the ROI were ranked by the software plugin. The pixels in the top 10%, which were presented on the same line, were selected. Among them, the pixels bearing higher intensity values that were equal to or greater than the sum of the background noise and median values were designated as target pixels (white); all other pixels were designated as null (black). The total percentage of these white pixels (gap) over the ROI length was automatically measured by the plugin. To obtain the total SI% value, the obtained total gap percentage was subtracted from 100%. Finally, the mean of the SI% values for each specimen was calculated. The data were subjected to normality analysis, and a nonparametric test was selected. The mean SI% values for each adhesive were statistically analysed by multiple comparisons using the Kruskal‒Wallis test. All analyses were performed using SPSS.

### Confocal laser scanning microscopy

After SS-OCT imaging, the cavities were cut perpendicularly to the flat surface using an IsoMet. The cut surface was polished under running water with silicon carbide paper (#600–2000 grit) to the location of the cross section captured by the imaging of SS-OCT, followed by diamond paste to 0.25 μm in grain size. Finally, the polished specimens were observed with CLSM (VK-X 150, Keyence, Tokyo, Japan) at 100 × and 400 × magnification levels.

## Results

### μSBS test

**Fig. 4:**
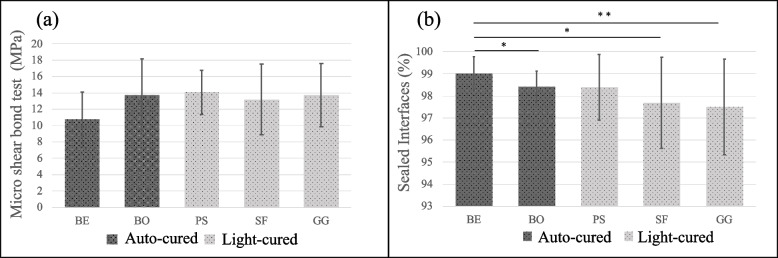
Graphs showing the results of the μSBS test and SS-OCT analysis. **a** Mean bond strength (MPa) and standard deviation resulting from the μSBS test (*n* = 20). No statistical significance was found among the groups. **b** Mean SI% and standard deviation resulting from the analysis of SS-OCT image (*n* = 16). Horizontal bars indicate significant differences between groups. (Kruskal‒Wallis test; significance at *p* < 0.05). (*… *p* < 0.05, **… *p* < 0.01)

**Fig. 5:**
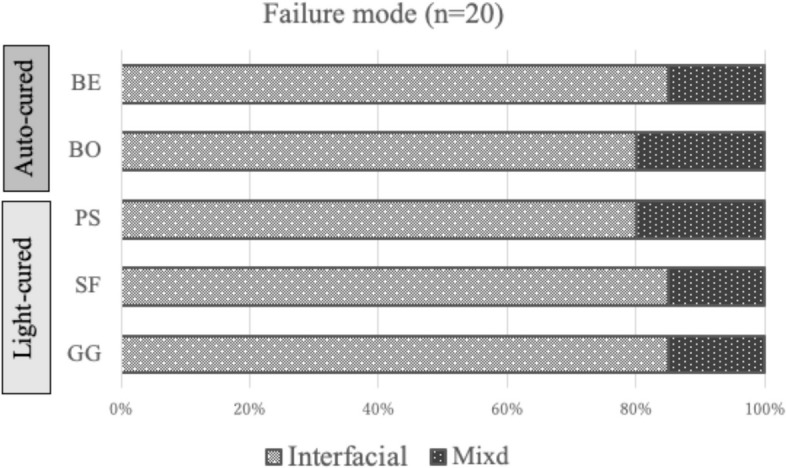
Percentage of failure modes (%)

**Fig. 6:**
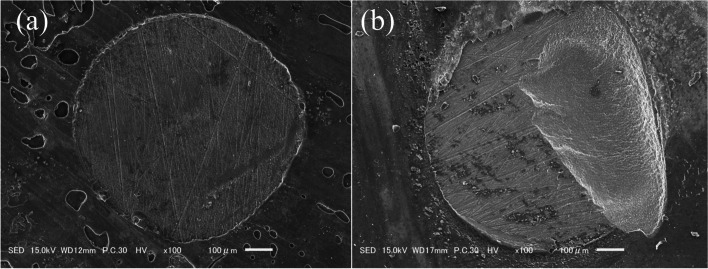
Representative SEM images of the failure modes. **a** Representative SEM image of an interfacial failure from the BE group. **b** Representative SEM image of a mixed failure from the BO group

The means and standard deviations of the μSBS values are shown in Table 2 and Fig. 4a. One-way ANOVA showed that the mean μSBS values were not significantly different among the five groups. The frequencies of failure modes and the results of the analysis are shown in Fig. 5. SEM images of representative failure modes are shown in Fig. 6. The results of the chi-square test showed no significant differences in the frequencies of failure modes among the five groups (*p* > 0.05). Most of the failure modes occurred at the bond interface between the bond and dentin.

**Table 2:** Values are mean (S.D.) (*n* = 20) in MPa

BE	10.75${ (3.34)}^{A}$
BO	13.76${(4.40)}^{A}$
PS	14.07${(2.71)}^{A}$
SF	13.20${(4.31)}^{A}$
GG	13.72${(3.88)}^{A}$

Identical capitalized letters in a column indicate the absence of statistically significant differences (one-way ANOVA; significance at *p*  < 0.05)

Interfacial: adhesive failure between the adhesive and dentin. Mixed: combination of the adhesive and cohesive failure modes. The chi-squared and Fisher’s exact test results showed no differences in frequencies between the five groups (*p* > 0.05). Most of the failure modes occurred at the bond interface between the bond and dentin.

### SS-OCT analysis

**Fig. 7:**
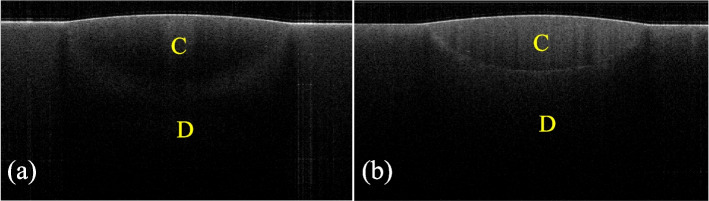
Representative SS-OCT images for observing marginal adaptation. C is the composite restoration, and D is dentin. **a** Representative SS-OCT image of an autocured bonding group (BE group). **b** Representative SS-OCT image of a light-cured bonding group (PS group)

**Fig. 8:**
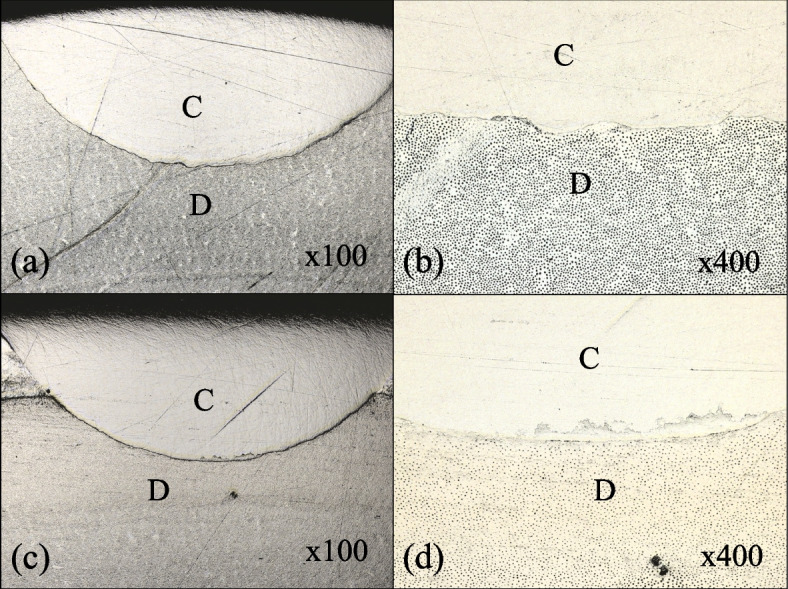
Representative CLSM images of marginal adaptation characteristics. C is the composite restoration, and D is dentin. **a** Representative CLSM image of an autocured bonding group (BO group) at 100 × magnification. **b** CLSM image of the sample shown in (**a**), observed at the bottom of the cavity at 400 × magnification. **c** Representative CLSM image of a light-cured bonding group (GG group) at 100 × magnification. **d** CLSM image of the sample shown in (**c**), observed at the bottom of the cavity at 400 × magnification

**Fig. 9:**
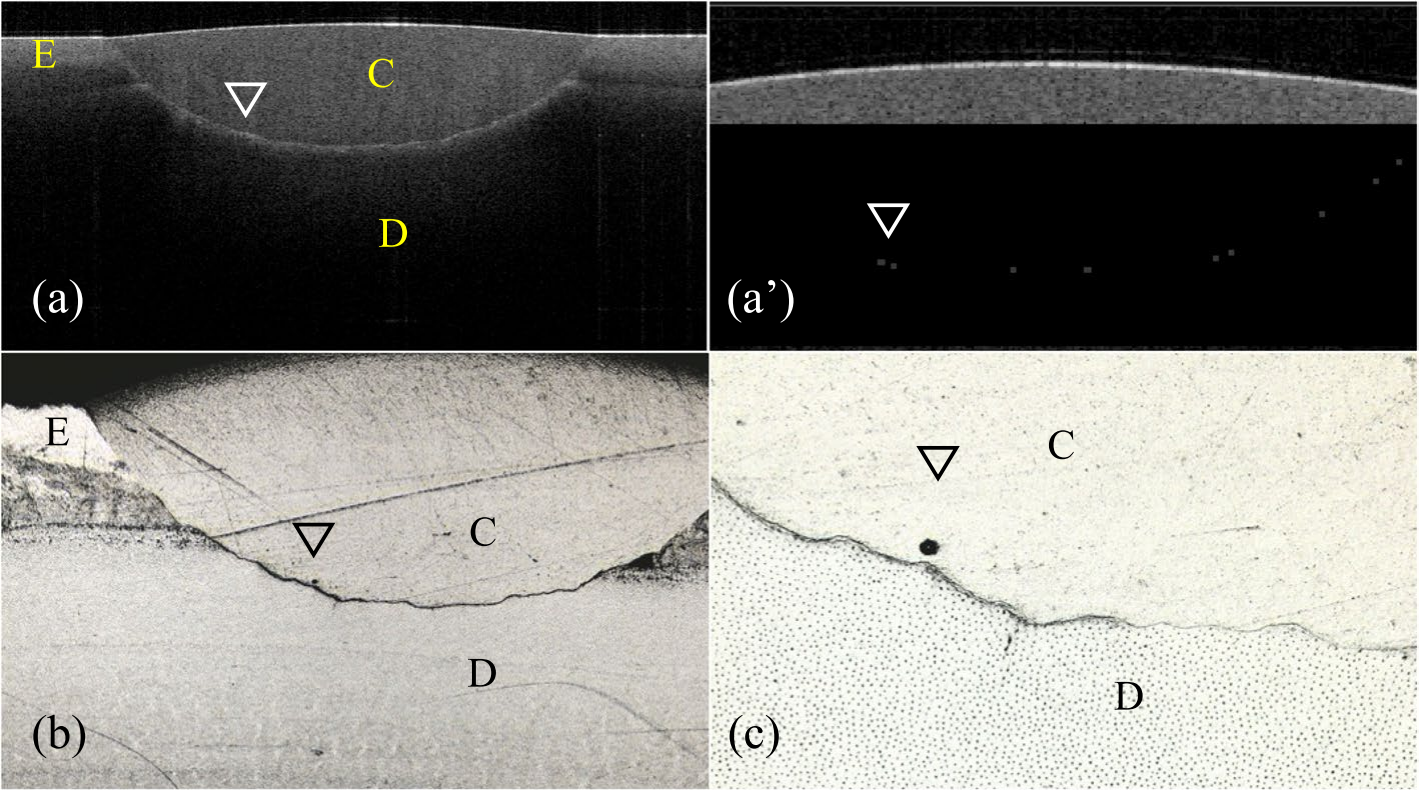
Representative SS-OCT and CLSM images when gaps are observed. E is enamel, C is the composite restoration, and D is dentin. Blank arrows denote the same areas on different images. **a** SS-OCT image showing increased brightness at the bottom of the cavity. **a**’ Binary image after applying the binarization process to the interfacial area. **b** CLSM image of a cross-section of a cavity of the sample in (**a**) at 100 × magnification. **c** CLSM image of the bottom of the cavity of the sample in (**a**) at 400 × magnification. The gap coincides with the brightness-increasing area shown in (**a**’)

A representative 2D SS-OCT image of the cavity is shown in Fig. 7. Representative confirmatory CLSM images are shown in Fig. 8. Figure 9 shows that the increase in signal intensity at the interface in the 2D OCT cross section corresponded to a gap in the confirmatory CLSM image. Table 3 and Fig. 4b show the results of SS-OCT analysis of SI% for each group. The mean SI% values and their standard deviations for all groups are plotted in Table 3. Conducting the Kruskal‒Wallis test on the OCT data revealed that groups with significantly different SI% values existed, rejecting the null hypothesis of this study (*p* < 0.05). The BE group of autocured bonding materials showed the highest mean SI% that significantly differed from those of the BO, SF, and GG groups. Although not significantly different, the mean SI% values of the BE and BO groups of the autocured bonding materials were higher than those of the light-cured bonding materials.

**Table 3:** Values are averages (S.D.) (*n* = 16)

BE	99.02${(0.76)}^{ABC}$
BO	98.43${(0.68)}^{A}$
PS	98.41${(1.49)}^{D}$
SF	97.69${(2.07)}^{B}$
GG	97.50${(2.17)}^{C}$

Identical capitalized letters in a column indicate a significant difference (Kruskal‒Wallis test; significance at *p* < 0.05)

## Discussion

In this in vitro study, we evaluated a new self-curing universal bonding material (Bondmer Lightless 2) and compared it with recently available light-curing bonding materials. μSBS tests were conducted on different adhesives and composite resins. The bond strength and failure modes of the specimens were evaluated using a universal testing machine and SEM, respectively. Additionally, marginal adaptation assessment was performed using SS-OCT and CLSM.

μSBS tests and SS-OCT imaging were performed to evaluate the bonding performance attributes of these adhesive systems. The results of the μSBS test showed no significant difference among the groups. In addition, previous papers have reported cohesive failure with increasing frequency as the bond strength increased in shear bond strength tests [27]. Similarly, no significant differences between each group were observed in the ratio of failure modes in this study. Therefore, the presence of a correlation between the bond strengths and failure modes in this study agreed with the correlations found in a previous study [27].

Although few studies using autocured adhesive systems in direct methods have been reported, scholars using autocured cements in indirect methods have shown that autocured cements have significantly lower bond strengths than light-cured cements [28–30]. Conventional autocured cements use a polymerization accelerator, such as a tertiary amine, and a polymerization initiator, such as a peroxide, and a polymerization reaction that mainly uses these agents has a slow polymerization speed due to the reaction of tertiary amines with acidic monomers reducing polymerization accelerators. The curing speed of dual-cured composite cements was 5–20 times slower during autocuring than during light-curing [31]. This phenomenon was why the adhesive performance of the autocured cement was lower than that of the light-cured cement, which could complete polymerization in a relatively short time in an environment with available light irradiation [32–34]. However, in this study, the bonding performance levels of the autocured adhesive systems were equivalent to those of the light-cured adhesive systems in the μSBS tests and failure mode observations. Similarly, in a previous paper using Bondmer Lightless as a bonding agent for dentin, the bond strength was reported to be equivalent to that of a light-curing bonding agent [35]. This disparity could be attributed to the incorporation of a borate catalyst in the new autocured universal adhesive agent, differing from conventional autocured adhesive agents. In contrast to tertiary amines, the borate catalyst reacted with an acidic three-dimensional self-reinforcing (3D-SR) monomer to form a boron compound. Subsequently, the boron was oxidized by the acidic monomer, generating a highly active polymerization initiator for chemical polymerization [36, 37]. This phenomenon resulted in an initial curing speed that was faster than that of a conventional chemically curing bonding agent, increasing degree of polymerization. This factor may be contributed to the equivalent adhesive performance demonstrated by the autocured bonding material, which was comparable to light-cured bonding materials.

As reported in a previous study [24], the conversion of light-cured bonding materials was affected by the irradiation time and the distance between the light curing unit and the cavity (the light intensity). Decreased light intensity resulted in insufficient polymerization, reducing the bond strength and marginal adaptation levels. With the methods of μSBS testing in this study, the cavity was not prepared to be deep, and the light irradiation time was conducted according to the manufacturer's instructions. Thus, the light intensity was supposedly sufficient for the adequate polymerization of the light-cured bonding materials. Nevertheless, no significant differences in the bond strengths and the failure modes existed between the group using the autocured bonding material and the group using the light-cured bonding material. Based on the results of this study, the amount of light reaching the bottom of the cavity decreased when the depth of the cavity increased without changing the irradiation time. Consequently, we anticipated that the light-cured bonding materials would exhibit lower bond strengths than those reported in this study.

Conversely, in terms of marginal adaptation, the BE group showed statistically significantly higher marginal adaptation levels than the BO, SF, and GG groups. Furthermore, the CLSM images corresponded with the gaps observed in the SS-OCT images. This result was consistent with that from a previous study [26] and with that from the SS-OCT marginal adaptation assessment in this study. However, no statistically significant differences between the light-cured bonding materials and the autocured materials were found in our μSBS test. This finding did not agree with the report [13], which suggested a correlation between marginal adaptation and bond strength in cavity evaluation by SS-OCT. The variations in the testing methods could impact the results; previous studies involved microtensile bond strength tests, which differ from the microshear bond strength test employed in this study. Moreover, in this study, the bond strengths of specimens with confirmed marginal adaptation were not evaluated, and the adhesive conditions differed between the SS-OCT assessment and μSBS test, potentially leading to different results relative to those from previous studies. To clarify the correlation between marginal adaptation and bond strength, it could be necessary to measure the bond strengths of specimens with confirmed cavity sealing in the future.

Due to the limitations of this study, the marginal adaptation levels of the BE and BO groups, which are autocured bonding materials, showed comparable or even higher values than those of light-cured bonding materials. This result suggested a potentially superior marginal adaptation for the autocured bonding materials. However, due to the relationship between bonding strength, it could be challenging to definitively state that autocured bonding materials exhibit superior marginal adaptation to light-cured bonding materials. Additionally, marginal adaptation was influenced by various factors, including the wettability and viscosity levels of composite resins; these characteristics could vary among different products. Further research would be necessary to investigate the impacts of these properties on marginal adaptation levels.

## Conclusions

In the bonding system using the new autocured bonding material, no significant difference in bond strength from the conventional one-step light-cured bonding material was present. In addition, the new autocured bonding material showed similar or even higher marginal sealing of the restoration than the conventional light-cured bonding material. This result indicated that the new autocured bonding material could be useful in clinical practice.

## Data Availability

The datasets used and analysed during this study are available from the corresponding author upon reasonable request.

## Abbreviations

1-SEA: One-step self-etching adhesives
2-SEA: Two-step self-etching adhesives
μSBS: Microshear bond strength
BO: Bondmer Lightless 2 with Estelite Bulk Fill Flow
BO: Bondmer Lightless 2 with a prototype composite resin
PS: Prime&Bond Universal with SDR flow +
SF: Scotchbond Universal with Filtek Bulk Fill
GG: G-Premio Bond with Gracefil BulkFlo
SEM: Scanning electron microscopy
OCT: Optical coherence tomography
SS-OCT: Swept-source optical coherence tomography
CLSM: Confocal laser scanning microscopy
Bis-GMA: Bisphenol A-glycidyl methacrylate
TEGDMA: Triethylene glycol dimethacrylate
HEMA: 2-Hyroxyethylmethacrylate
MTU-6: 6-Methacryloyloxyhexyl 2-thiouracil-5-carboxylate
Bis-MPEPP: 2,2-Bis[(4-methacryloxy polyethoxy) phenyl] propane
UDMA: Urethane dimethacrylate
BisEMA: Ethoxylated bisphenol-A dimethacrylate
Bis-MEPP: Bismethacrylic acid isopropylidenebis (p-phenyleneoxyethylene) ester
ROI: Region of interest
SI%: Sealed interface percentage
3D-SR: Three-dimensional self-reinforcing
